# Acute pyelonephritis including an overlooked renal cell carcinoma 

**DOI:** 10.5414/CNCS107906

**Published:** 2013-07-02

**Authors:** Soo Kyeong Song, Ji Soo Song, Hong Pil Hwang, Sik Lee

**Affiliations:** 1 Department of Internal Medicine,; 2 Radiology, and; 3 General Surgery, Research Institute of Clinical Medicine, Chonbuk National University Medical School, Jeonju, Republic of Korea

**Keywords:** pyelonephritis, renal cell carcinoma

## Abstract

Hidden renal mass is not evident during severe inflammation such as acute pyelonephritis, pyonephrosis, or renal abscess. Deceptive inflammatory presentations that can occur in aggressive synchronous renal malignancies can significantly delay the diagnosis of cancer. Herein, we report a case of acute pyelonephritis involving overlooked renal cell carcinoma.

## Introduction 

Renal neoplasm is a common occurrence that is easily diagnosed using routine imaging [1]. But renal malignancies associated with inflammatory diseases, such as renal abscess and pyelonephritis, can lead to a delay in the detection of cancer. Severe inflammatory diseases of the kidney have been uncommonly reported to be associated with renal malignancies, particularly xanthogranulomatous pyelonephritis [2, 3]. The systemic signs of inflammation and malignancy can be mixed, and imaging studies sometimes cannot distinguish between these two diseases [4], leading to misinterpretation in standard imaging procedures. In contrast, lesions can mimic a renal malignancy on imaging confirmed normal or on benign renal tissue after surgical resection – these are referred to as renal pseudotumors. We describe a case of acute pyelonephritis, including an overlooked renal cell carcinoma. 

## Case report 

**Figure 1:**
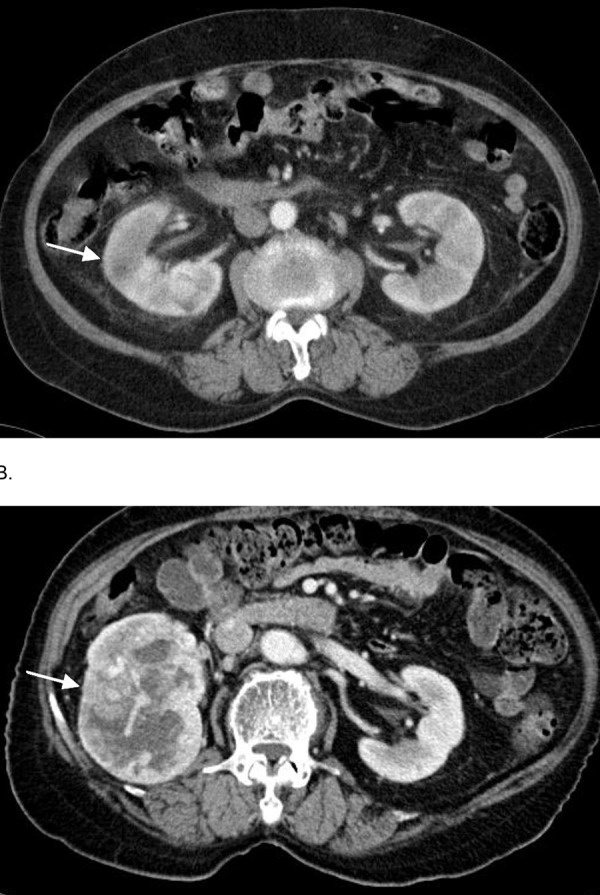
Figure 1. A: Computed tomography (CT) of the abdomen and pelvis demonstrated some hypoenhancing lesions with perinephric strandings in both kidneys and a suggestive small abscess in the right kidney (arrow), which was compatible with acute pyelonephritis. B: 5 years after the treatment of acute pyelonephritis, CT of abdomen and pelvis indicated an exophytic 6 × 8 cm sized mass with necrosis in the mid and lower portion of the right kidney (arrow).

A 76-year-old woman was transferred to our hospital for evaluation of a right renal mass incidentally detected at a local clinic. She had a past history of acute pyelonephritis, having been treated in our hospital 5 years prior. At that time, she visited our hospital with a 10-day history of high fever and chills. On physical examination, a physician elicited pain with percussion at both costovertebral angles. Laboratory findings were hemoglobin, 11.3 g/dl; white blood cells, 9.2 × 10^3^/l (polymorphoneuclear leukocyte, 7.1 × 10^3^/l; lymphocyte, 1.3 × 10^3^/l; monocyte, 9.2 × 10^3^/l); platelets, 164 × 10^3^ /l; blood urea nitrogen, 9 mg/dl; creatinine, 1.0 mg/dl; and high sensitivity-C reactive protein, 260 mg/l. Urinalysis showed many erythrocytes per high power field with 21 – 25 leukocytes per high power field. Multidetector computed tomography (MDCT) of the abdomen and pelvis showed some hypoenhanced lesions with perinephric strandings in both kidneys and a suggestive small abscess in the right kidney, which was compatible with acute pyelonephritis (Figure 1A). The culture of urine grew Escherichia coli that were susceptible to cefazolin. She improved clinically after antibiotic treatment and was discharged in a healthy state. There was no follow-up because of the lack of urinary symptoms and complaints. 

Five years after the treatment of acute pyelonephritis, she visited a local clinic with the complaint of right flank discomfort. Right renal mass and multiple nodules in both lower lung fields were detected on local MDCT, prompting her referral to our hospital. MDCT of abdomen and pelvis demonstrated an exophytic 6 × 8 cm sized mass with necrosis in the mid and lower portions of the right kidney (Figure 1B). Chest CT revealed randomly distributed multiple nodules in both lung fields and filing defect by thrombi in the left main pulmonary artery as well as the interlobar artery, which suggested hematogenous lung metastasis and pulmonary thromboembolism (data not shown). Laparoscopic retroperitoneal radical nephrectomy was performed, and the histopathologic results disclosed the clear cell type of renal cell carcinoma. She has been taking sorafenib tosilate and warfarin, and has been followed for 12 months post-operatively with no evidence of relapse. 

## Discussion 

Renal malignancies are uncommonly associated with inflammatory diseases such as renal abscess, pyelonephritis, and xanthogranulomatous pyelonephritis; these can lead to a delay in the detection of cancer. Kidney infection is easily diagnosed with a symptom, physical examination, and urinalysis. But, the systemic signs of inflammation and malignancy can be mixed, and imaging studies sometimes cannot distinguish between these two diseases, leading to misinterpretation in standard imaging procedures [4]. 

Symptoms of acute pyelonephritis include pain or burning during urination, an urgent need to urinate, fever, hematuria, and nausea with or without vomiting. Radiologic evaluation is warranted for patients with complicated pyelonephritis and pyelonephritis who also have symptoms of renal colic or a history of renal stones, diabetes, infection with a particularly virulent organism, a history of prior urologic surgery, immunosuppression, repeated episodes of pyelonephritis, or urosepsis. CT scan and ultrasonography are useful modalities. CT without contrast has become the standard radiographic study for demonstrating calculi, gas-forming infections, hemorrhage, obstruction, and abscesses. Contrast is needed to demonstrate alterations in renal perfusion. CT features of pyelonephritis show a focal wedge-shaped area of low attenuation, due to ischemia induced by marked neutrophilic infiltration, and edema, without a well defined wall around it and without an overlying bulge on the renal surface, which distinguishes it from renal cell carcinoma. However, because some infiltrative renal tumors may have an appearance similar to that of focal pyelonephritis, extension of the acute inflammatory process into the perirenal soft tissues may give the appearance of a renal malignancy. As well, sonography or CT findings of renal abscess reveal a well-defined heterogenous mass that at times may simulate a renal malignancy [1]. In these conditions, renal malignancy has been confirmed after malignancy progression or metastasis. Therefore, because the prognosis of renal malignancy is very different from pyelonephritis, the possibility of renal malignancy should always be kept in mind and followed up in imaging study, even if atypical imaging findings are present, to avoid delaying the proper diagnosis and treatment. 

## Conclusion 

This case emphasizes that physicians should consider severe inflammatory diseases, such as acute pyelonephritis, in the differential diagnosis of indefinable renal malignancies with a high index of suspicion. Since recognition of this rare disease entity could prevent delays in diagnosis and treatment, follow-up of medical imaging is required for patients with atypical clinical presentations, for those who fail to respond to conventional therapy, and for the diagnosis of acute severe pyelonephritis and its complications, which can also include abscess formations. 

## Conflict of interest

None declared.
